# Application-Oriented Chemical Optimization of a Metakaolin Based Geopolymer

**DOI:** 10.3390/ma6051920

**Published:** 2013-05-10

**Authors:** Claudio Ferone, Francesco Colangelo, Giuseppina Roviello, Domenico Asprone, Costantino Menna, Alberto Balsamo, Andrea Prota, Raffaele Cioffi, Gaetano Manfredi

**Affiliations:** 1 Department of Technology, University of Naples “Parthenope”, Centro Direzionale, Is. C4, Napoli 80143, Italy; E-Mails: francesco.colangelo@uniparthenope.it (F.C.); giuseppina.roviello@uniparthenope.it (G.R.); rcioffi@uniparthenope.it (R.C.); 2 Department of Structures for Engineering and Architecture, University of Naples “Federico II”, Naples 80125, Italy; E-Mails: d.asprone@unina.it (D.A.); costantino.menna@unina.it (C.M.); alberto.balsamo@unina.it (A.B.); aprota@unina.it (A.P.); gaetano.manfredi@unina.it (G.M.)

**Keywords:** geopolymers, drying shrinkage, EB-FRP, metakaolin

## Abstract

In this study the development of a metakaolin based geopolymeric mortar to be used as bonding matrix for external strengthening of reinforced concrete beams is reported. Four geopolymer formulations have been obtained by varying the composition of the activating solution in terms of SiO_2_/Na_2_O ratio. The obtained samples have been characterized from a structural, microstructural and mechanical point of view. The differences in structure and microstructure have been correlated to the mechanical properties. A major issue of drying shrinkage has been encountered in the high Si/Al ratio samples. In the light of the characterization results, the optimal geopolymer composition was then applied to fasten steel fibers to reinforced concrete beams. The mechanical behavior of the strengthened reinforced beams was evaluated by four-points bending tests, which were performed also on reinforced concrete beams as they are for comparison. The preliminary results of the bending tests point out an excellent behavior of the geopolymeric mixture tested, with the failure load of the reinforced beams roughly twice that of the control beam.

## 1. Introduction

The term “geopolymer” was first used by J. Davidovits in the late 1970s and nowadays identify a family of amorphous alkali or alkali-silicate activated aluminosilicate binders of composition M_2_O·*m*Al_2_O_3_·*n*SiO_2_, usually with *m* ≈ 1 and 2 ≤ *n* ≤ 6 (M usually is Na or K) [1].

The synthesis of geopolymers takes place by polycondensation reactions of metakaolinite (kaolinite calcined at 600–700 °C) or many natural and artificial silico-aluminates with alkali metal (Na or K) hydroxide and/or silicate [2,3,4,5,6,7,8,9,10]. When in contact with a high pH alkaline solution, aluminosilicate reactive materials are rapidly dissolved into solution resulting in the release of aluminate and silicate ions, most likely in the monomeric form, which afterwards condensate to form a rigid network. Amorphous geopolymers are obtained by carrying out the polycondensation reaction at temperatures from 20 to 90 °C, while crystalline materials are formed in the autoclave at higher temperatures, up to 200 °C [1]. Crystalline products form at low temperatures, too, if low modulus silicate solutions are used [11].

Geopolymer based materials are attractive because excellent mechanical properties, high early strength, high durability, freeze-thaw resistance, low chloride diffusion rate, abrasion resistance, thermal stability and fire resistance, can be achieved [12,13,14,15,16]. Due to their lower Ca content, they are more resistant to acid attack than Portland cement based materials [17]. In addition, geopolymer based materials are of great interest because of the reduced energy requirement for their manufacture. In fact, the reaction pathway requires either metakaolinite or raw silico-aluminates so that greenhouse gas emission can be reduced up to 80% in comparison to traditional cement-based materials [15,18]. In fact, even if natural aggregates are substituted by sustainable artificial ones, manufactured by using industrial wastes [19,20,21], the production of CO_2_ in concrete industry is mainly linked to the employment of Portland cement as a binder.

Applications of geopolymer-based materials in the fields of new ceramics, binders, matrices for hazardous waste stabilization, fire-resistant materials, asbestos-free materials, and high-tech materials have been documented [22,23,24,25,26,27].

Among the possible applications of geopolymers, their use as bonding matrices in fiber reinforced structural applications could be highly beneficial. In the last decades, the need for structural rehabilitation of existing reinforced concrete (RC) and masonry structures pushed the scientific community to conduct extensive research on externally bonded, fiber reinforced organic polymers (EB-FRP) applications, investigating the feasibility of this retrofit technique for a variety of loading conditions, for several structural elements, and in various environmental conditions. Current EB-FRP applications represent a well-established technique for the rehabilitation of existing structures, considered by international codes and guidelines [28,29] as a proper and valid option for structural retrofitting. However, research in FRP community is still ongoing, dealing with a number of concerns on EB-FRP applications. Namely, the mechanical degradation of EB-FRPs with temperature is the weakest point of EB-FRPs, restricting the field of application only to conditions where temperatures higher than the resin glass transition temperature *Tg* are not experienced [30,31,32,33]. Unfortunately, *Tg* values of commonly used matrices for FRP are in the range 60–80 °C [28]. Therefore, FRP performance is of particular concern, especially for fire conditions, also considering the flame spread and the toxic fumes evolution of combusting polymer matrices. To deal with this issue a large number of research activities have been conducted. Studies about the high temperature mechanical stability of EB-FRPs highlight how epoxy adhesive temperature in excess of 60–70 °C produces a loss integrity between the EB-FRP and concrete [34,35]. Fire insulating schemes for the application of FRPs or new polymeric matrices with higher values of *Tg* have been extensively investigated as well [36,37,38], but research in this direction has not yet produced significant results. For these reasons, all the existing codes and guidelines do not account for FRP contribution to the resistance of the retrofitted structural elements in case of fire conditions.

A further advantage of geopolymers compared to epoxy adhesives is related to their inorganic silico-aluminate nature, which makes these materials more similar to the concrete support from a chemical and physical point of view.

The current use of geopolymer resins in EB-FRP applications is not fully developed due to technical issues that characterize these applications. In fact, so far, good mechanical and physical properties of geopolymer composite systems have been obtained by controlling the curing conditions in terms of high temperature and/or controlled pressure [39].

Hence, a long term investigation was undertaken to evaluate the suitability of a metakaolinite based geopolymer as bonding matrix for external strengthening of reinforced concrete beams [40]. Metakaolin was chosen as starting material because of its purity and high reactivity.

Several works are present in the literature about the optimization of metakaolin based geopolymers and the relationship between geopolymer composition (mainly the Si/Al ratio) and their mechanical, physical and microstructural properties [41,42], but these papers refer to curing conditions involving a thermal treatment at temperatures higher then room temperature. Curing conditions sensibly affect the final properties of a geopolymer [43,44], and the results obtained at high temperature may not be valid at room temperature [45]. Moreover, it is often difficult to control the temperature when geopolymers are prepared/applied out of a laboratory.

In order to simulate the actual operative conditions, the formulation of a metakaolin based geopolymer mixture to be used at room temperature, without any fine control of the temperature and humidity, has been the object of the present paper.

A preliminary application study was performed, too. The obtained mixtures have been tested in EB-FRP applications with mono directional steel fiber fabrics to strengthen reinforced concrete beams.

## 2. Experimental Section

### 2.1. Source Materials

The metakaolin powder, provided by Neuchem, has the following composition: Al_2_O_3_ 41.90 wt %; SiO_2_ 52.90 wt %; K_2_O 0.77 wt %; Fe_2_O_3_ 1.60 wt %; TiO_2_ 1.80 wt %; MgO 0.19 wt %; CaO 0.17 wt %; specific surface area 12.69 m^2^/g (data provided by the producer) and d_50_ = 3.64 μm, as determined by particle size distribution analysis performed by a Malvern Mastersizer 3000 laser particle size analyzer.

The alkaline activating solutions were prepared by using NaOH in pellets (Baker, analytical R grade) and two sodium silicate solutions supplied by Prochin Italia S.r.l. with the composition: Solution No. 1—SiO_2_ 27.40 wt %; Na_2_O 8.15 wt %; H_2_O 64.45 wt %; Solution No. 2—SiO_2_ 29.50 wt %; Na_2_O 14.70 wt %; H_2_O 55.80 wt %.

A pure quartz powder provided by Sibelco (MILLISIL SA12, SiO_2_ 99 wt %; maximum particle diameter 63 μm; specific surface area 0.35 m^2^/g) was added as filler to the metakaolin and the activating solution to prepare the final adhesive mortar.

A high-strength, mono-directional steel fiber fabric (MAPE WRAP S fabric) supplied by Mapei, was used as strengthening material. Table 1 summarize the main properties of the steel external reinforcement used.

**Table 1:** Properties of steel external reinforcement.

Properties	Value
Cord coating	Zinc
Number of filaments per cord	38
Cord area (mm^2^)	1.78
Cords per m	210
Tensile strength (MPa)	2845
Elongation at breakage (mm/mm)	0.0135
Tensile Modulus of Elasticity *E*_f_ (GPa)	210

### 2.2. Optimization of the Geopolymer Matrix

#### 2.2.1. Specimens Preparation

The compositions of geopolymers have been formulated to ensure that the Al/Na ratio is constant at 1, providing sufficient alkali to enable complete charge balancing of the negatively charged tetrahedral aluminum centers. Sodium silicate solutions characterized by molar SiO_2_/Na_2_O ratio (*r*) of 0, 0.67, 1.34 and 1.67, and molar H_2_O/Na_2_O ratio of 10.5, were prepared by using a 10 M NaOH solution (*r* = 0), adding a 15 M NaOH solution to the sodium silicate solution No. 1 (*r* = 0.67), or dissolving solid sodium hydroxide into the sodium silicate solution No. 1 or No. 2 (*r* = 1.34 and *r* = 1.67, respectively). This resulted in a total of four different specimens compositions with nominal chemical composition (assuming that geopolymerization was completed) Na_2_O·(SiO_2_)*z*·Al_2_O_3_·10.5H_2_O, where *z* is 2.15, 2.80, 3.50 and 3.80. From this point onward, the four mixtures will be coded as 1.07, 1.40, 1.75 and 1.90, respectively, according to their Si/Al ratio.

The main parameters of the prepared mixtures are summarized in Table 2.

**Table 2:** Synoptic table of the prepared mixtures.

**sample**	**1.07**	**1.40**	**1.75**	**1.90**
*r*	0	0.67	1.34	1.67
**Chemical composition**	Na_2_O·(SiO_2_)_2.15_·Al_2_O_3_·(H_2_O)_10.5_	Na_2_O·(SiO_2_)_2.80_·Al_2_O_3_·(H_2_O)_10.5_	Na_2_O·(SiO_2_)_3.50_·Al_2_O_3_·(H_2_O)_10.5_	Na_2_O·(SiO_2_)_3.80_·Al_2_O_3_·(H_2_O)_10.5_
**Mix Design (g)**	–	–	–	–
Sodium Silicate	–	60 (No. 1)	120 (No. 1)	139 (No. 2)
Solid NaOH	–	–	20	6.3
NaOH (aq)	110 (10 M)	64 (15 M)	–	–
Metakaolin	100	100	100	100

Alkaline activating solutions were prepared 24 h prior to be mixed with metakaolin. The polycondensation mixture was homogenized in a Hobart mixer for 10 minutes. The fresh mixes were of fluid consistence and were easily poured into a series of cylindrical polyethylene molds of size *d* × *h* = 3 cm × 6 cm. The molded samples were cured for seven days at room temperature, keeping the lids closed to ensure 100% relative humidity. Afterwards, samples were transferred from the sealed vessels into open vessels and kept at room temperature without any fine control of humidity. Their behavior has been monitored for greater than 6 months.

#### 2.2.2. Specimens Characterization

The obtained specimens were subjected to Unconfined Compressive Strength (UCS) determination by using a 100 kN capacity Controls MCC8 testing machine. Compressive strength determinations were carried out 7 days (at the end of the curing phase) and 28 days after the specimens’ preparation.

Samples characterization was performed also by mineralogical, spectroscopic and thermal analyses, mercury intrusion porosimetry and Scanning Electron Microscopy (SEM) observations.

The mineralogical analysis (X ray diffraction analysis, XRD) was performed by means of a Philips PW 1730 diffractometer (CuKα radiation, 5°–60° 2θ range, step width 0.02° 2θ and 1 s data collection per step).

Spectroscopic analysis was carried out by using Fourier Transform Infra-Red Analysis (FT-IR). FT-IR absorption spectra were recorded in the 4000–400 cm^−1^ range using a Nicolet system, Nexus model, equipped with a DTGS KBr (deuterated triglycine sulfate with potassium bromide windows) detector. A spectral resolution of 2 cm^−1^ was chosen. 2.0 mg of each test sample was mixed with 200 mg of KBr in an agate mortar, and then pressed into pellets of 13 mm diameter. The spectrum of each sample represents an average of 32 scans.

Thermal analysis was performed by means of Thermogravimetric analysis (TGA) making use of a TA Instrument SDT2960 (weight of the sample: 10 mg; heating rate: 10 °C min^−1^; atmosphere: air) and Differential Scanning Calorimetry (DSC) making use of a Mettler Toledo DSC822E (weight of the sample: 4 mg; heating rate: 10 °C min^−1^; atmosphere: nitrogen).

Mercury Intrusion Porosimetry was performed by using two porosimeters, namely Thermo Pascal 140 and Thermo Pascal 440, which operate at a maximum pressure of 400 kPa and 400 MPa, respectively.

SEM analysis was carried out by means of a FEI Quanta 200 FEG microscope. EDS analyses were performed by using Energy Dispersion Spectrometer Oxford Inca Energy System 250 equipped with INCAx-act LN2-free detector at 20 kV voltage.

## 3. Results and Discussion

**Figure 1:**
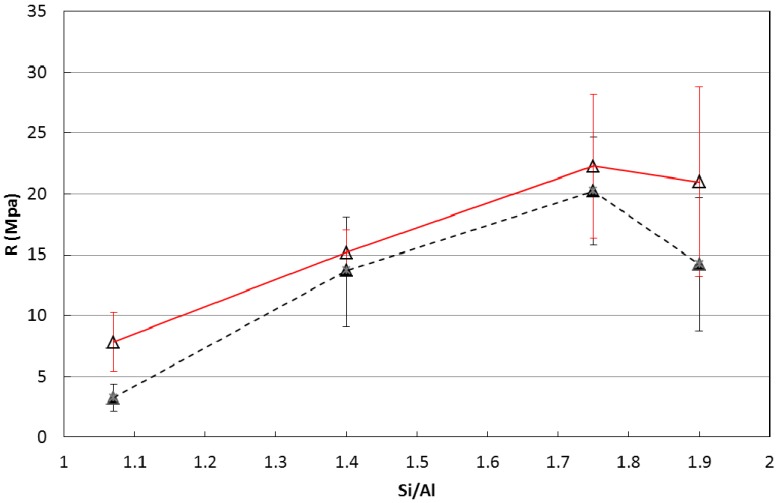
Unconfined compressive strength (R) of cylindrical specimens as a function of the Si/Al ratio at 7 days (black dotted curve) and 28 days (red curve). Error bars indicate the average deviation from the mean over the three samples measured.

Figure 1 shows the results of the UCS determinations at 7 and 28 days. All values presented in the current work are an average of three samples with error reported as average deviation from mean.

The compressive strengths of the specimens at 7 and 28 days are quite similar, providing evidence of a rapid strength development, typical of geopolymer based materials. The general trend confirms literature findings [42], in fact the strength increases as Si/Al ratio increases. Nonetheless some discrepancies are evident: the absolute strength values are considerably lower than those reported by Duxson *et al*. and the maximum strength occurs at Si/Al = 1.75 instead of 1.90. The shape and dimensions of the specimens were similar, so these differences may be likely due to the different curing conditions, either in terms of curing temperature, 40 °C [42] *vs*. room temperature (current work), or in terms of humidity conditions, sealed vessels [42] *vs*. open air (current work). It should be noted that specimens 1.90 showed evident fracture lines at 28 days, likely due to drying shrinkage. The higher drying shrinkage of the specimen characterized by the highest Si/Al ratio is in good agreement with recently published data [45] and is related to the curing at room temperature. This behavior seems somewhat in contrast with that reported in [14] related to specimens cured at 40 °C for 24 h and kept in sealed vessels. These authors found a better dimensional stability of specimens with high Si/Al ratios than those with low one. This seeming inconsistence, besides the different curing conditions, is due to the different test performed. In [14] the authors evaluated the thermodilatometric behavior of the hardened and cured samples under continuous heating, whereas our results are obtained considering the shrinkage at room temperature.

**Figure 2:**
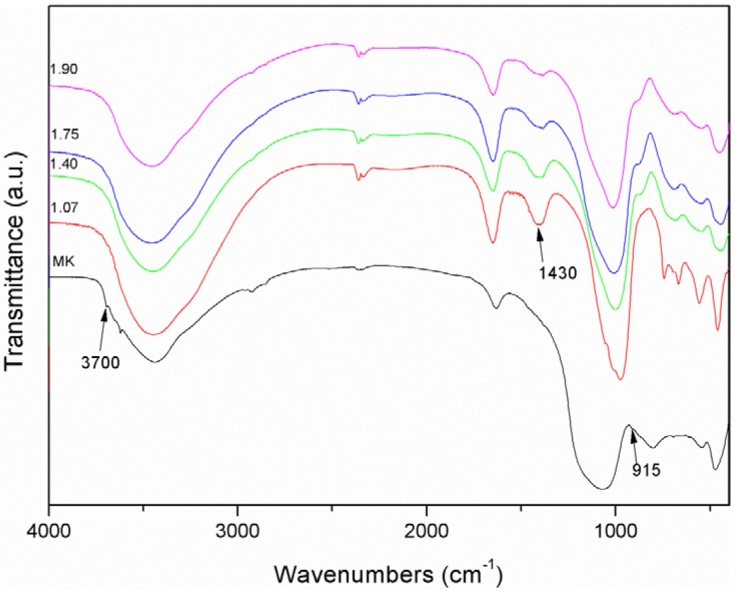
IR spectra of metakaolin (MK), and of samples 1.07, 1.40, 1.75 and 1.90.

The FT-IR spectra of the original metakaolin and of the geopolymer mixtures prepared are shown in Figure 2. Spectra were acquired after 7 days of curing and 21 days of exposure to laboratory ambient conditions.

The analysis of the metakaolin spectrum (Figure 2) provides evidence that the conversion from kaolin to metakaolin was not complete, as the absorption bands at 3700 cm^−1^, in the –OH stretching region, and at 915 cm^−1^, ascribed to the bending vibrations of hydroxyl groups associated with Al cations in kaolinite [46], even if extremely broad, are clearly visible. The presence of residual kaolinite in the precursor could, at least in part, explain the low strength values exhibited by the geopolymer specimens [47]. An absorption band at about 1430 cm^−1^, related to Na_2_CO_3_ [48], is present in all spectra of geopolymer samples. Its presence is related to unreacted NaOH, which was carbonated by atmospheric CO_2_. The presence of Na_2_CO_3_ is rather common in geopolymer samples, whatever the ratio between the constituents [2]. Nonetheless, the intensity of this absorption peak is substantially more pronounced for 1.07 and 1.40 samples than for 1.75 and 1.90. Since nominal Al/Na ratio is equal to 1 for all mixtures, this fact evidences that, as mixtures 1.07 and 1.40 were produced from activating solutions with SiO_2_/Na_2_O < 1, the lower availability of Si in solution leads to a reduction in Al incorporation into the geopolymeric structure [49] of these two mixtures. The different polymer structure could explain also the lower strength values of the specimens with Si/Al 1.07 and 1.40. Furthermore, free alkalis may interact with a cement-based support and could be the cause of weathering phenomena. The analysis of the FTIR spectra of the mixtures 1.40, 1.75 and 1.90 provides evidence of a shift towards lower wavenumbers of the peak at about 1000 cm^−1^, related to the Si–O asymmetric stretching [48]. This shift is a typical consequence [50] of the geopolymeric reaction and is related to a reorganization of the Si environment. The 1.07 FT-IR spectrum at wavenumbers lower than 1000 cm^−1^ is completely different from that of the other samples, as it shows the presence of sharp and intense peaks in the range 600–800 cm^−1^ related to Si–O–Al vibrations [48,51]. This pattern evidences that this sample has a different structure with respect to the other three and is characterized by the presence of crystalline, presumably zeolitic, phases.

**Figure 3:**
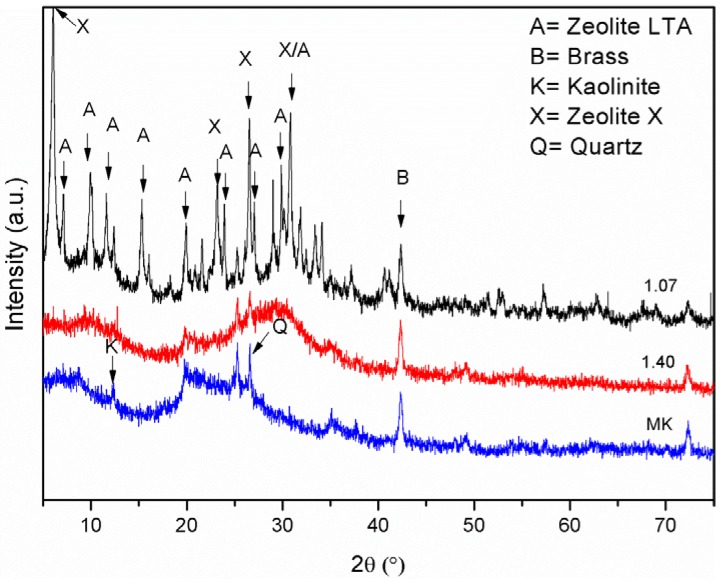
XRD patterns of metakaolin (MK) and of samples 1.07 and 1.40.

In order to confirm the real nature of sample 1.07, an XRD diffraction analysis has been carried out. Figure 3 shows the XRD patterns of the original metakaolin, of the sample 1.07 and of the sample 1.40 (XRD patterns of the samples 1.75 and 1.90 are not shown as they closely resemble that of sample 1.40). One broad diffraction peak that can be attributed to kaolinite (JCPDS card No. 14-164) is detectable in the XRD pattern of metakaolin, together with a peak at 42°–3° 2θ that can be attributed to the brass (JCPDS card No. 50-1333) used for the sample holder. The broad peak at 15°–35° 2θ is characteristic of the metakaolin structure [52]. The 1.07 XRD pattern shows the presence of many diffraction peaks which can be reasonably attributed to zeolite LTA (JCPDS card No. 73-2340) and zeolite X (JCPDS card No. 38-237), thus confirming that this sample is not a proper geopolymer, as shown through the FT-IR results. Crystallization of zeolitic phases from alkaline activation of metakaolin was reported in the literature [53,54,55], at higher temperatures. At room temperature, the formation of an amorphous solid or hydroxysodalite was reported [11,54]. An explanation of the different behavior of the sodium hydroxide activated system with respect to those activated by sodium silicate starts from the observation that the binder gel formed in geopolymerization is likely to contain many nanometer-sized zeolitic crystals [56]. The formation of highly crystalline zeolitic phases, instead of amorphous geopolymers containing zeolites nanocrystals, was explained by considering that the nucleation rate of solid products immediately surrounding the dissolving aluminosilicate source particles was higher in the presence of soluble silicates than in the presence of only hydroxide. Therefore, a lower number of nuclei have the possibility to grow into larger crystals. On the other side, the 1.40 XRD pattern shows the typical broad “hump” centered at approximately 27°–29° 2θ, which can be considered the distinguishing feature of the diffractogram of any geopolymer [56].

Thermal analysis of geopolymer based materials is useful to evaluate the amount and the type of water held by the sample. Total water content of the geopolymer samples, as determined by TGA (not reported), ranges between 23.3% and 28.4%. These values are lower than values usually found in the literature [45] because in the current work thermal analysis was performed after 21 days exposure to the laboratory atmosphere, instead of 100% RH [45].

**Figure 4:**
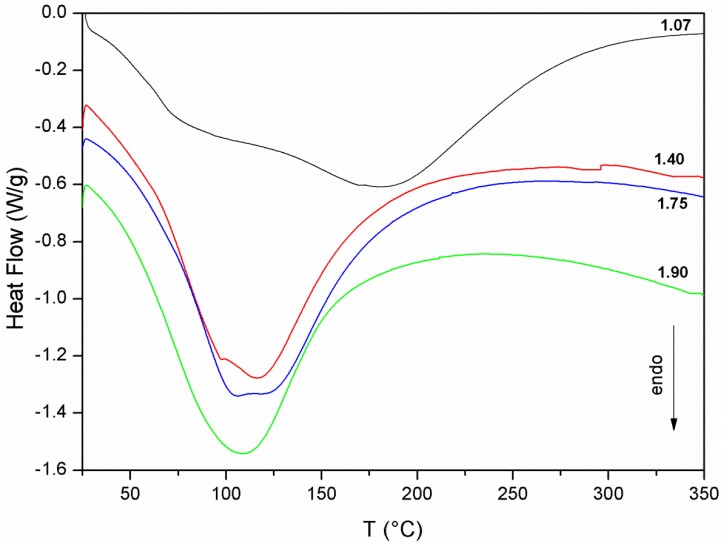
DSC curves of samples 1.07, 1.40, 1.75 and 1.90.

More interesting is the analysis of the DSC curves of the four samples (Figure 4).

The DSC signals show a first endothermic peak at temperatures lower than 100n °C, which can be attributed to the evaporation of “free” water [57,58], and an endothermic shoulder which becomes more evident as the Si/Al ratio decrease. This endothermic shoulder is due to the evaporation of “zeolitic” [59] or “interstitial” [58] water. This type of water is associated with activating and extra-framework cations in the form of solvation water. The attribution of this second endothermic peak to zeolitic water is facilitated in the case of sample 1.07, where the presence of zeolitic phases was distinctly detected by the other characterization techniques adopted. Zeolitic water plus hydroxyl water can be considered as “nonevaporable” water at ambient conditions, thus their total amount must not be accounted for, dealing with the drying shrinkage. Evaporable water is related to the parameter W:

W = 100 – (ΔW%_125–800_/ΔW%_tot_) × 100 (1)

where: ΔW%_125–800_ is the percentage mass loss of the samples in the temperature range 125–800 °C, as determined by thermo gravimetric analysis, and ΔW%_tot_ is the total initial water content, as determined by considering the water added in the preparation of the samples. The residual mass variation at temperatures up to 800 °C has been considered to evaluate also the possible “hydroxyl” water.

**Figure 5:**
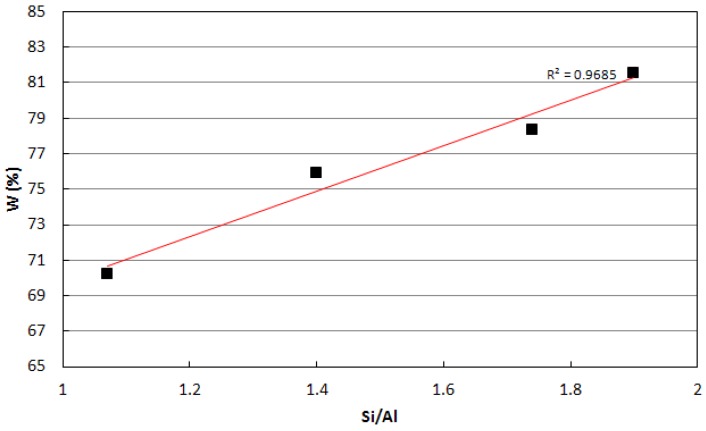
W parameter of samples 1.07, 1.40, 1.75 and 1.90.

Figure 5 shows the variation of the parameter W *vs*. the Si/Al ratio. It is evident that the percentage of evaporable water linearly increases with the Si/Al ratio. The higher percentage of evaporable water of the samples characterized by higher Si/Al ratios accounts for their higher sensitivity to drying shrinkage.

**Figure 6:**
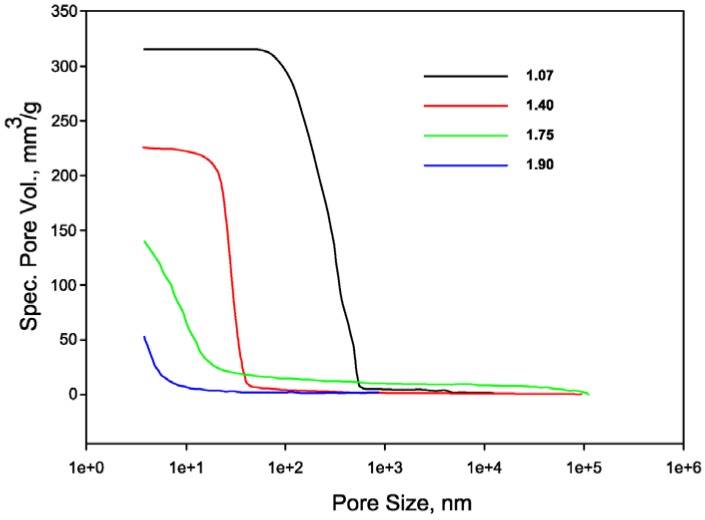
Pore size distribution of geopolymer samples.

This higher sensitivity to drying shrinkage is related also to the microstructure of the high silicon geopolymer. It is characterized by a low total porosity, constituted only of micro- and meso-pores as reported in Figure 6. Figure 6 shows also that the total specific pore volume of the specimens decreases as the Si/Al ratio increases. This trend is in good agreement with the values of the apparent density of the specimens, which are as follows (expressed in g/cm^3^): d_1.07_ = 1.33, d_1.40_ = 1.40, d_1.75_ = 1.49, d_1.90_ = 1.63.

Therefore, geopolymers with higher Si/Al ratios could be more prone to drying shrinkage owing to higher capillary strains, related to the evaporation of the water, than samples with low Si/Al ratios characterized by pores of higher dimensions. The higher elastic modulus of geopolymers with higher Si/Al ratios [60] may also play a negative role, inducing high internal stresses into the specimen and the consequent macroscopic fracture.

**Figure 7:**
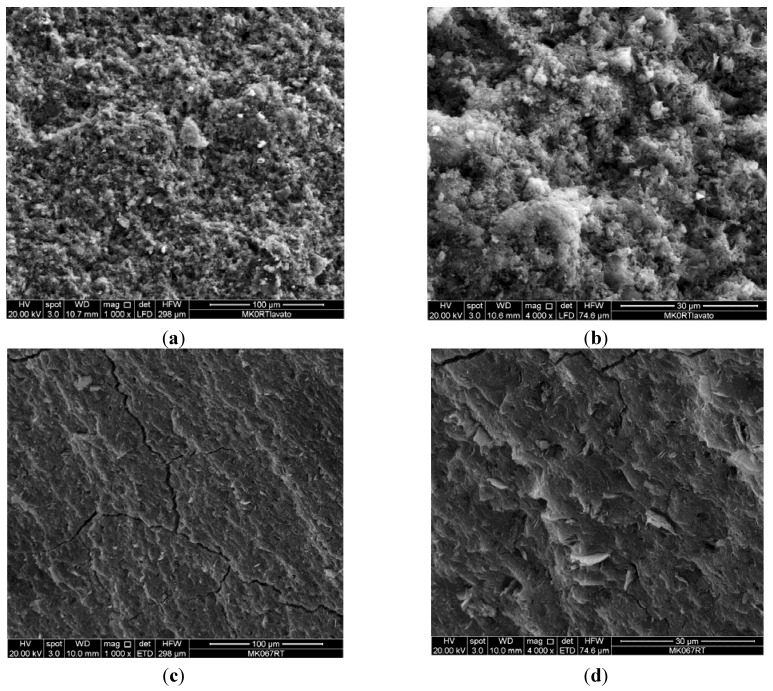

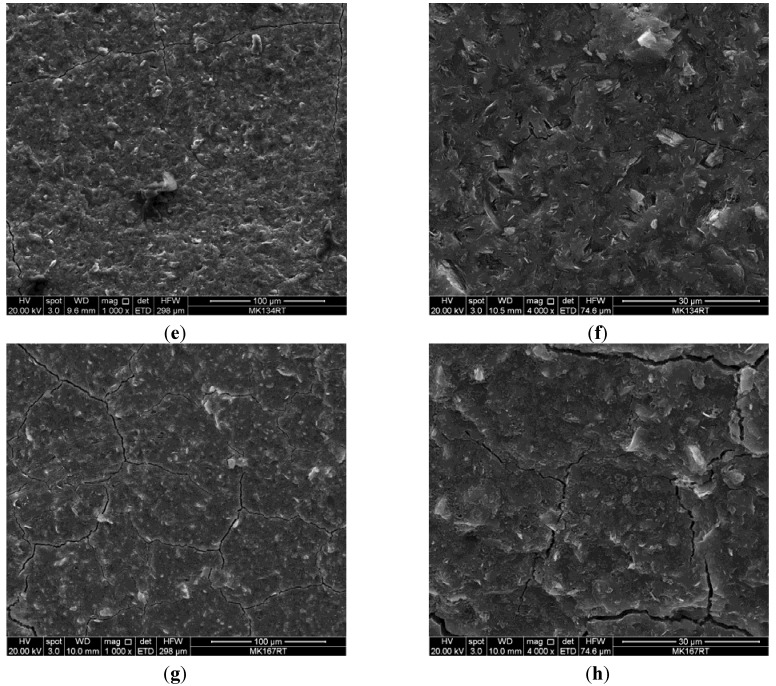
(**a**,**b**) SEM micrographs of samples 1.07; (**c**,**d**) 1.40; (**e**,**f**) 1.75; (**g**,**h**) 1.90.

SEM micrographs of freshly obtained fracture surfaces of the four geopolymers studied exhibit significant change in microstructure with variation of Si/Al ratio (Figure 7). The change in microstructure appears more dramatic between samples 1.07 and 1.40. Specimen 1.07 doesn’t show evident fracture lines at low magnification (Figure 7a) but exhibits a scarcely compact and porous structure (Figure 7b). All the specimens with higher Si/Al ratios exhibit an evident fracture network, especially samples 1.40 and 1.90 (Figure 7c,g). The extensive microfracture network of sample 1.40 doesn’t induce a macroscopic fracture of the specimen likely because the porosity is characterized by macropores and the elastic modulus is lower than that of sample 1.90. At a higher magnification, the structure of samples 1.75 and 1.90 is characterized by the presence of a dense and glassy matrix containing unreacted metakaolin particles (Figure 7f,h).

**Figure 8:**
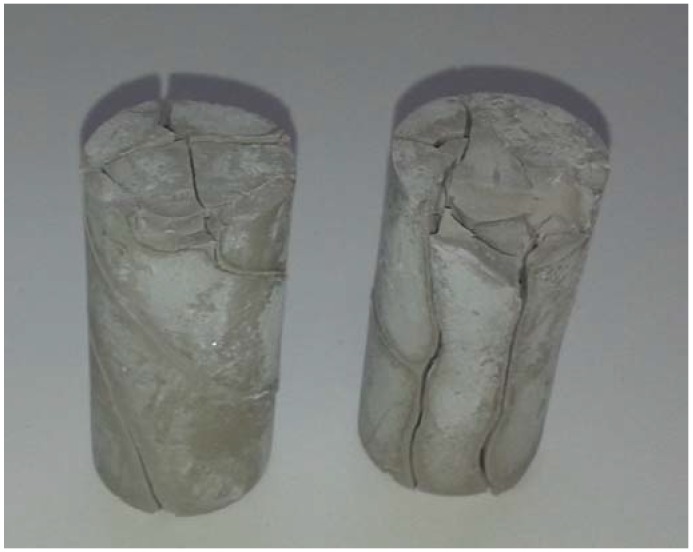
Specimens 1.90 after 40 days exposure to ambient conditions.

At 40 days from mixture casting specimens 1.90 were completely destroyed, as showed in Figure 8.

The above discussions and observations determined the choice of the mixture 1.75 as the most proper for the application as EB-FRP bonding matrix.

## 4. Application

### 4.1. Optimization of the Geopolymeric Adhesive

Samples 1.75 showed some macrocracks on its external surface at 120 days from casting. In order to avoid possible fractures and also to reduce the binding matrix amount, an unreactive filler in the form of fine quartz powder was added. A filler characterized by small particle size was chosen in order to ensure the complete impregnation of the reinforcement fabrics. The filler was added in 1:1 weight ratio with the metakaolin powder. As regard the flow characteristics, the obtained mortar has a smooth, plastic quality and is easily spread with a trowel. Mix design of the geopolymer mortar is: Sodium silicate = 800 kg/m^3^; Solid NaOH = 133 kg/m^3^; Metakaolin = 667 kg/m^3^; Quartz powder = 667 kg/m^3^.

The obtained mortar was used to prepare cubic samples of 5 cm edge, according to ASTM C109/C109M-11b. In the light of the above discussed results, cubic specimens have been wrapped in a PVC film and cured at room temperature in order to avoid excessive early water evaporation. UCS determinations were performed at 7 and 28 days and the results are the following: R (7 days) = 57 ± 1 MPa; R (28 days) = 99 ± 1 MPa (expressed as the average of three determinations).

In this case, compressive strength measurements are much higher than those reported in Figure 1 for geopolymer pastes and are comparable, if not greater, than those reported in the literature [59]. The substitution of 50% of metakaolin with a stiff and high strength material such as fine quartz powder, was expected to considerably increase the strength of the system [61]. The addition of an inorganic fine filler, which can be approximately considered as a material with no porosity, has the primary effect of reducing the total porosity of the specimens. In fact, fine quartz powder can fill the interstitial space inside the skeleton of the hardened microstructure of the pastes, which led to more dense structure, thus compressive strengths increased. Furthermore, the partial replace of the metakaolin powder with quartz powder reduced the water content of the specimens and, consequently, the drying shrinkage was also reduced. In fact, no shrinkage phenomena were observed on the external surfaces of the specimens even at long observation times. The inert filler acted as a rigid skeleton, whereas curing the specimens wrapped with the PVC film avoided the early evaporation of the water.

### 4.2. Application of the Geopolymer Based Reinforcing System

The mortar described in the previous section was used in combination with a high strength monodirectional steel fabric and applied to reinforced concrete beams of dimensions 400 mm × 200 mm × 3800 mm for strengthening purposes. Two samples were considered, whereas one beam was not strengthened and used as control. The beams were tested under four-point monotonic loading in order to determine their ultimate load-carrying capacity.

**Figure 9:**
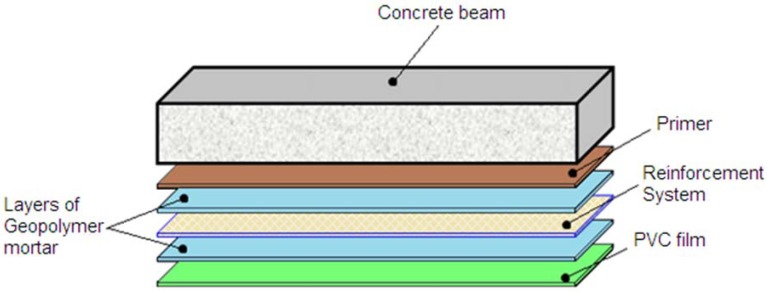
EB-FRP system layers.

The reinforcing system, 200 mm wide, was applied over the middle span, with a total length of 3.10 m, ensuring that it would not extend beyond the supports. Before the application of the strengthening system, the bottom face of all beams was mechanically scratched and cleaned to ensure proper bond of the reinforcement. First, a geopolymer primer was applied to avoid the drying of the geopolymeric mortar by capillary absorption at the geopolymer-concrete interface. The primer consisted of a geopolymer mixture of identical composition as the matrix, without inorganic filler and with a higher water content. The chemical composition of the primer can be expressed as Na_2_O·(SiO_2_)_3.50_·Al_2_O_3_·13.3H_2_O. After one day, the strengthening system was applied to the beams according the following procedure: (i) the geopolymer mixture was prepared by using a high shear mixer to mix the components of the resin for a total of 3 min and a first geopolymer layer was applied on the concrete surface; (ii) the steel fabric was cut according to design length and pressed onto the wet geopolymer layer ensuring a full impregnation of the fabric; (iii) an additional layer of geopolymer matrix was laid on the reinforcement. Finally, a PVC film was placed surrounding the last geopolymer layer and maintained for the total curing period to avoid water evaporation. All the layers are depicted in Figure 9. Good workability was observed for about 20–30 min after mixing the geopolymer components. No mechanical anchorage system was used to fix the external reinforcement to the beam.

**Figure 10:**
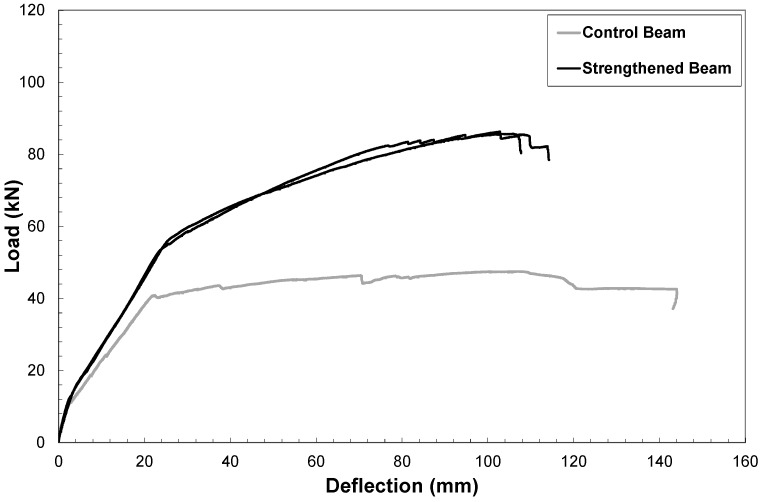
Flexural stress-strain curves of strengthened beams and control beam.

All the specimens were allowed to harden and cure at room conditions for 28 days. The flexural strength determinations were performed by a 150 kN hydraulic actuator and the test was carried out under displacement control. During the tests, the midspan deflection of the beam was measured by means of three vertical linear displacement gauges, which provided average displacement measurements used to derive the force-deflection diagram showed in Figure 10.

The strengthened system exhibited good results in terms of flexural performance. Both reinforced beams showed a very similar trend in terms of load-deflection curves. After yielding of the internal steel of rebars, the slope of the force-deflection curve is higher than that of the control beam, due to the increasing tensile force in the external steel reinforcement. For both specimens, steel cord failure occurred at a load of 85.7 kN and 86.3 kN by increasing the ultimate load capacity of the beams by about 100%. In particular, close to failure, small drops in the load deflection curve are caused by the progressive brittle failure of single steel cords within the external reinforcement. No debonding between geopolymer matrix and RC substrate was observed.

**Figure 11:**
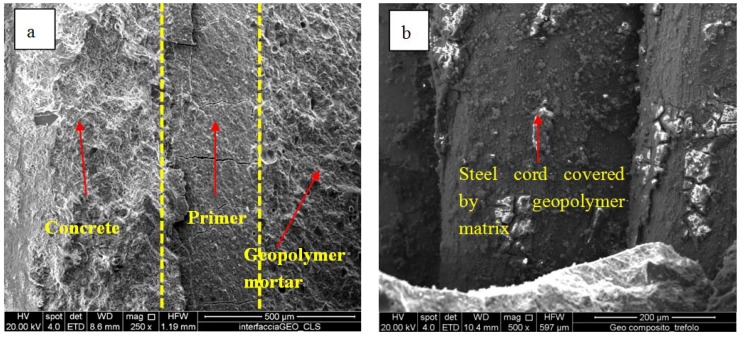
(**a**) SEM micrographs of a sample of geopolymeric mortar detached from the concrete support; (**b**) a steel cord covered by a geopolymer matrix layer.

This feature is mainly due to the geopolymer matrix which is able to form a strong and continuous bond between the surface of the concrete and the fiber reinforcement, verified by two SEM micrographs showed in Figure 11a,b.

In particular, Figure 11a shows a sample of geopolymeric mortar detached from the concrete support after the flexural strength test. Three distinct zones are evident: the concrete support, the geopolymer primer layer and the geopolymer mortar, with no evident discontinuity among the three phases. Figure 11b shows a steel cord embedded in the geopolymer matrix. This sample was taken from the reinforcing system at the end of the flexural strength test, too. It is evident that the steel fiber is completely covered by the geopolymer matrix. The mortar layer has detached from the steel cord and the fracture happened inside the mortar rather than at the interface with the steel cord, proving a strong interfacial interaction. This result is also confirmed by literature data [62] and by very preliminary tests the authors conducted on the investigated geopolymer, where steel molds were used to cast the mixture. In fact, these tests demonstrated such an exceptional bond between the steel walls of the molds and the geopolymer matrix to make the molds themselves unusable.

## 4. Conclusions

The aim of this paper was to develop a metakaolin based geopolymer to be used as EB-FRP bonding matrix. The development of the matrix was made by considering four compositions of the geopolymer in terms of Si/Al ratio and by characterizing the obtained materials. EB-FRP bonding matrices require geopolymer curing treatments to be conducted at ambient conditions.

The main conclusions which can be drawn from the results of this work are the following:

- Curing of metakaolin based geopolymers at ambient conditions is critical because a substantial material shrinkage can occur. This issue is related to the amount of evaporable water of the sample, which was found to increase with the Si/Al ratio of the geopolymer mixture.
- A medium-high value of Si/Al ratio = 1.75 was found to be the best compromise between mechanical performances and shrinkage issues. Nonetheless, the addition of a fine quartz powder as filler and the control of the early water evaporation were found to be crucial in obtaining the best performances.
- The results of preliminary tests conducted by employing the mortar as matrix of a composite with long steel fibers to strengthen reinforced concrete beams were greatly encouraging as the ultimate load capacity of the beams increased more than 100% with respect to the plain beam and no debonding between geopolymer matrix and concrete substrate was observed.

Further developments of this system will regard the optimization of the rheological behavior by possibly obtaining a thixotropic one, in view of application to ceiling or vertical surfaces, the use of other kinds of fibers and the comparison with traditional cement based matrices.
